# Supplementary material for: Exocyst-mediated membrane trafficking of the lissencephaly-associated ECM receptor dystroglycan is required for proper brain compartmentalization
Source: eLife. 2021 Feb 23;10:e63868. doi: 10.7554/eLife.63868 (PMC7929561; doi:10.7554/eLife.63868)
Supplement: Supplementary file 1. — *Misguided = fused β lobes or β lobe neurons projecting into γ lobe space. **Underdeveloped = smaller α or β lobes due to α lobe neurons projecting into β lobe space or vice versa. aCompared to Oregon (Control). bCompared to DgO86/+ (Control). cCompared to DgO86/Sec6Ex15. dCompared to DgO86/Sec10f03085. eCompared to DgO86/Sec151. For comparison of the observed phenotypes, two-way tables and χ2 test were used. [file elife-63868-supp1.docx]

**Supplementary File 1. MB Morphology of is affected by deregulation of Dg and the exocyst**

| *Genotype* | Observed MB phenotypes | | | p-value | Number of lobes analyzed |
| --- | --- | --- | --- | --- | --- |
|  | **Normal** | **Misguided** | **Under-developed** |  |  |
| *Oregon* (*Control*) | 91% | 4% | 4% |  | 70 |
| *Dg^O86^* | 49% | 20% | 31% | ^a^p=8.2E^-7^ | 55 |
| *Dg^O86^/ Dg^O55^* | 39% | 29% | 32% | ^a^p=4.5E^-8^ | 38 |
| *insc>Dg* | 8% | 54% | 38% | ^a^p=1.8E^-13^ | 24 |
| *201Y>Dg* | 45% | 21% | 34% | ^a^p=1.8E^-7^ | 47 |
| *201Y>Dg^RNAi^* | 36% | 36% | 29% | ^a^p=4.2E^-6^ | 14 |
| *c305a>Dg* | 78% | 11% | 11% | ^a^p=0.163 | 37 |
| *c305a> Dg^RNAi^* | 76% | 10% | 14% | ^a^p=0.077 | 42 |
| *Dg^O86^/+ (Control)* | 81% | 0% | 19% | ^a^p=0.033 | 32 |
| *Dg^O86^/Sec5^E10^* | 58% | 26% | 16% | ^b^p=9.2E^-3^  ^c^p=0.139  ^d^p=0.021  ^e^p=0.060 | 19 |
| *Dg^O86^/Sec6^Ex15^* | 31% | 41% | 28% | ^b^p=1.5E^-5^  ^d^p=0.480  ^e^p=0.031 | 39 |
| *Dg^O86^/Sec10^f03085^* | 20% | 40% | 40% | ^b^p=1.98E^-6^  ^e^p=0.052 | 30 |
| *Dg^O86^/Sec15^1^* | 36% | 16% | 48% | ^b^p=3.0E^-4^ | 44 |
| *Sec15^1^*/*Sec5^E10^* | 31% | 47% | 22% | ^a^p=1.5E^-5^ | 32 |
| *Sec15^1^*/*Sec6^Ex15^* | 26% | 21% | 53% | ^a^p=4.0E^-4^ | 38 |
| *Sec5^E10^*/ *Sec10^f03085^* | 35% | 38% | 28% | ^a^p=6.1E^-5^ | 40 |
| *Sec15^1^*/*Sec6^KG08199^* | 20% | 7% | 73% | ^a^p=9.0E^-7^ | 44 |

*** --** Misguided = fused β lobes or β lobe neurons projecting into γ lobe space

**** --** Underdeveloped = smaller α or β lobes due to α lobe neurons projecting into β lobe space or vice versa

^a^ – compared to *Oregon* (*Control*), ^b^ – compared to *Dg^O86^/+ (Control),* ^c^ – compared to *Dg^O86^/Sec6^Ex15^,*

^d^ – compared to *Dg^O86^/Sec10^f03085^,* compared to *Dg^O86^/Sec15^1^*

For comparison of the observed phenotypes, two-way tables and χ^2^-test were used.
