# Supplementary material for: Exocyst-mediated membrane trafficking of the lissencephaly-associated ECM receptor dystroglycan is required for proper brain compartmentalization
Source: eLife. 2021 Feb 23;10:e63868. doi: 10.7554/eLife.63868 (PMC7929561; doi:10.7554/eLife.63868)
Supplement: Supplementary file 2. [file elife-63868-supp2.docx]

**Supplementary File 2. Dg-interacting proteins in neurons**

| Gene Symbol | Protein name | FlyBase ID | Cellular locali-zation | Molecular function | Human homo-log(s) | Disease association/ Risk factors |
| --- | --- | --- | --- | --- | --- | --- |
| *Dg* | Dystroglycan | FBgn0034072 | Membrane-associated receptor | ECM receptor, Dystrophin-Glycoprotein complex component | DAG1 | Muscular dystrophies, Dystroglycanopathies |
| *PlexA* | Plexin A | FBgn0025741 | Membrane associated receptor | Protein binding, Axon guidance receptor, Heparin binding, Semaphorin receptor, GTPase activator | PLXNA2 | Cardiac hypertrophy |
| *boss* | bride of sevenless | FBgn0000206 | Membrane associated receptor | Transmembrane receptor protein tyrosine kinase activator, G-protein coupled receptor, sevenless binding | GPRC5B | Body mass index, Obesity-related traits |
| *CG10226* | CG10226 | FBgn0035695 | Membrane-associated receptor | Drug transmembrane transporter, ATPase | ABCB1 ABCB4 ABCB5 ABCB11 | Fasting plasma glucose, Metabolic syndrome, Cholestasis, progressive familial intrahepatic 2, Response to statin therapy, Gallbladder disease 1, Dental caries |
| *CG1090* | CG1090 | FBgn0037238 | Membrane-associated | Calcium, potassium:sodium antiporter, Transmembrane ion transport | SLC24A1-5 | Skin pigmentation, Eye and hair color, Matrix metalloproteinase levels, Pulmonary function decline, Cognitive performance, Night blindness |
| *kcc* | kazachoc | FBgn0261794 | Membrane-associated | Amino acid transmembrane transporter, Potassium:chloride symporter | SLC12A6 | Agenesis of the corpus callosum with peripheral neuropathy |
| *CG5853* | CG5853 | FBgn0032167 | Membrane associated | ABC transporter, ATPase activity, coupled to transmembrane movement of substances | ABCG2 | Cardiovascular disease risk factors, Dental caries, Urate levels, Lipoprotein-associated phospholipase A2 activity change in response to statin therapy |
| *Rme-8* | Receptor mediated endocytosis 8 | FBgn0015477 | Membrane associated | Notch Receptor mediated endocytosis | IPO11 | Nemaline Myopathy Achalasia-Addisonianism-Alacrima Syndrome |
| *uzip* | unzipped | FBgn0004055 | Membrane  associated | cell adhesion molecule, regulation of axon guidance | SYAP1 | Robinow Syndrome |
| *eys* | eyes shut | FBgn0031414 | ECM | Extracellular matrix structural constituent, Calcium ion binding | EYS  CRB1 AGRN | Retinitis pigmentosa, Asthma, Leber congenital amaurosis, Pigmented paravenous chorioretinal atrophy, Myasthenia, limb-girdle, familial |
| *Cpr66D* | Cuticular protein 66D | FBgn0052029 | ECM | Structural constituent of chitin-based cuticle | ZNF160 | No disease terms found |
| *igl* | igloo | FBgn0013467 | Cyto-skeleton | Myosin light chain binding, Calmodulin binding | NRGN | Schizophrenia |
| *CalpA* | Calpain-A | FBgn0012051 | Cyto-skeleton | calcium-dependent modulator, C protease | GMPS | No disease terms found |
| *vimar* | visceral mesodermal armadillo-repeats | FBgn0022960 | Cytosol | Ral GTPase binding | RAP1  GDS1 | Biochemical measures, Lymphocytic leukemia |
| *MESK2* | Misexpres-sion suppressor of KSR 2 | FBgn0043070 | Cytosol | Positive regulation of Ras protein signal transduction | NDRG1 NDRG4 | Charcot-Marie-Tooth disease, Electrocardiographic traits, QT interval |
| *Cp190* | Centrosomal protein 190kD | FBgn0000283 | Cytosol | Chromatin insulator sequence binding, POZ domain binding, Homodimerization, Microtubule binding, Metal ion binding, DNA binding | MDN1 | No disease terms found |
| *Apoltp* | Apolipoprotein lipid transfer particle | FBgn0032136 | Cytosol | Lipoprotein particle receptor binding, Lipid binding, Lipid transporter | APOB | Cardiovascular disease risk factors, Erectile dysfunction and prostate cancer treatment, Hypertriglyceridemia, Metabolic syndrome, Hypercholesterolemia, Hypobetalipoproteinemia |
| *faf* | fat facets | FBgn0005632 | Cytosol | Thiol-dependent ubiquitin-specific protease, Protein binding | USP9Y USP24 | Spermatogenic failure, Metabolite levels |
| *bchs* | blue cheese | FBgn0043362 | Cytosol | Metal ion binding. | WDFY4 | Response to antidepressant treatment, Systemic lupus erythematosus |
| *garz* | garten-zwerg | FBgn0264560 | Cytosol | Guanyl-nucleotide exchange factor, ARF guanyl-nucleotide exchange factor | GBF1 | No disease terms found |
| *eIF-5A* | eIF-5A | FBgn0285952 | Cytosol | Translation regulator, Translation initiation factor, Translation elongation factor, Ribosome binding | EIF5A | No disease terms found |
| *msi* | musashi | FBgn0011666 | Cytosol | mRNA binding, Translation repressor, Nucleic acid binding | MSI1-2 | No disease terms found |
| *Cul3* | Cullin 3 | FBgn0261268 | Cytosol | Ubiquitin protein ligase binding, Ubiquitin-protein transferase | CUL3 CUL4B | Pseudohypoaldosteronism type IIB, Mental retardation |
| *Cul4* | Cullin 4 | FBgn0033260 | Cytosol | Ubiquitin protein ligase binding | CUL4B | Mental retardation |
| *MP1* | Melanization Protease 1 | FBgn0027930 | Cytosol | Serine-type peptidase | No ortholog found | - |
| *CG9780* | CG9780 | FBgn0037230 | Cytosol | Metalloendopeptidase | KEL PHEX | F-cell distribution, Immune response to smallpox, Tourette syndrome, Hypophosphatemic rickets |
| *CG4538* | CG4538 | FBgn0038745 | Cytosol | Serine-type endopeptidase, Unfolded protein binding, ATP binding, Proteolysis, Protein folding | CLPX | No disease terms found |
| *CSN4* | COP9 signalosome subunit 4 | FBgn0027054 | Cytosol | COP9 signalosome component, NEDD8-specific protease, Transcription regulatory region DNA binding, Protein binding. | CSN4 | No disease terms found |
| *CG6453* | GCS2β, Glucosidase 2 β subunit | FBgn0032643 | Cytosol  ER | Alpha-1,4-glucosidase, N-glycan processin | PRKCSH | Polycystic liver disease |
| *dor* | deep orange | FBgn0000482 | Cytosol | Zinc ion binding, Syntaxin binding | VPS18 | Visceral adipose tissue/subcutaneous adipose tissue ratio |
| *Pka-R1* | Protein kinase, cAMP-dependent, regulatory subunit type 1 | FBgn0259243 | Cytosol | cAMP-dependent protein kinase regulator, cyclic nucleotide binding | PRKAR1A | Acrodysostosis 1, Adrenocortical tumor, Carney complex, Myxoma intracardiac, Pigmented nodular adrenocortical disease, Thyroid carcinoma, Adverse response to lamotrigine and phenytoin |
| *CG32850* | CG32850 | FBgn0052850 | Cytosol | Zinc ion binding, Ubiquitin-protein transferase | RNF11 | Ventricular conduction |
| *CG9372* | CG9372 | FBgn0036891 | Cytosol | Serine-type peptidase, Serine-type endopeptidase | PRSS12 | Mental retardation |
| *CG31205* | CG31205 | FBgn0051205 | Cytosol | Serine-type endopeptidase | F11 | Venous thromboembolism, |
| *Pka-C1* | Protein kinase, cAMP-dependent, catalytic subunit 1 | FBgn0000273 | Cytosol | cAMP-dependent protein kinase, Protein serine/threonine kinase, ATP binding, Protein binding | PRKACB | Breast cancer (male) |
| *Pkc98E* | Protein C kinase 98E | FBgn0003093 | Cytosol | Diacylglycerol binding, ATP binding, Protein kinase C, Protein serine/threonine kinase, Calcium-dependent protein kinase C | PRKCE  PRKCF | Hematocrit, Red blood cell traits, Rheumatoid arthritis, Cerebral infarction, Ischemic stroke |
| *for* | foraging | FBgn0000721 | Cytosol | Cyclic nucleotide-dependent protein kinase, ATP binding, Protein serine/threonine kinase, cGMP-dependent protein kinase | PRKG1 | Asthma, Obesity-related traits, Height, HIV-1 susceptibility, Pubertal anthropometrics |
| *PI4KIIIα* | Phosphatidylinositol 4-kinase III α | FBgn0267350 | Cytosol | Phosphatidylinositol 4-kinase | PI4KA | No disease terms found |
| *CG1332* | CG43367 | FBgn0263110 | Cytosol | Sphingomyelin phosphodiesterase activator | NBEAL1 NBEAL2 | Gray platelet syndrome, Obesity-related traits |
| *inaD* | inactivation no after-potential D | FBgn0001263 | Cytosol | Calmodulin binding, Myosin binding, Receptor signaling complex scaffold | INADL | Obesity-related traits |
| *Srp54k* | Signal recognition particle protein 54k | FBgn0010747 | Cytosol | Signal sequence binding, 7S RNA binding, GTPase activity | SRP54 | No disease terms found |
| *CG3689* | CG3689 | FBgn0035987 | Cytosol | mRNA binding; hydrolase activity | NUDT21 | No disease terms found |
| *Rrp46* | Rrp46 | FBgn0037815 | Cytosol | 3'5' exoribonuclease, mRNA processing, Regulation of gene expression | EXOSC5 | No disease terms found |
| *RanGAP* | Ran GTPase activating protein | FBgn0003346 | Cytosol | GTPase activator | RANGAP1 | No disease terms found |
| *Crag* | Calmodulin-binding protein related to a Rab3 GDP/GTP exchange protein | FBgn0025864 | Cytosol | Rab guanyl-nucleotide exchange factor, Calmodulin binding, Guanyl-nucleotide exchange factor, Rab GTPase binding | DENND4A DENND4B | Red blood cell traits, Lentiform nucleus volume |
| *CG3626* | CG3626 | FBgn0029706 | Cytosol | Oxidoreductase, Pyruvate dehydrogenase phosphatase regulator | PDPR | No disease terms found |
| *Hem* | HEM-protein | FBgn0011771 | Cytosol | Protein binding | NCKAP1L | Obesity-related traits |
| *CG13185* | CG13185 | FBgn0033661 | Cytosol | ATPase activity, ATP binding | MDN1 | No disease terms found |
| *CG4729* | CG4729 | FBgn0036623 | Cytosol | 1-acylglycerol-3-phosphate O-acyltransferase | AGPAT3 | Phospholipid levels |
| *CSN5* | COP9 signalosome subunit 5 | FBgn0027053 | Cytosol | Protein binding, NEDD8-specific protease | COPS5 | No disease terms found |
| *Lin19* | Cul1,  Cullin 1 | FBgn0015509 | Cytosol | Ubiquitin protein ligase binding | CUL1 | No disease terms found |
| *Uev1A* | Ubiquitin-conjugating enzyme variant 1A | FBgn0035601 | Cytosol | Ubiquitin protein ligase, Ubiquitin conjugating enzyme binding | UBE2V2 | No disease terms found |
| *bur* | burgundy | FBgn0000239 | Cytosol | GMP synthetase | OSBPL9 | No disease terms found |
| *eIF2B-α* | eIF2B-α | FBgn0039726 | Cytosol  ER | Enzyme regulator, Translation initiation factor | EIF2B1 | Ovarioleukodystrophy, Leukoencephalopathy with vanishing white matter |
| *CG11306* | CG11306 | FBgn0037108 | Cytosol | alpha-1,2-mannosyltransferase, N-terminal, Glycosyl transferase | ALG11 | Congenital disorder of glycosylation, type I |
| *eEF1α2* | eukaryotic translation elongation factor 1 alpha 2 | FBgn0000557 | Cytosol | translation elongation factor | No ortholog found | No disease terms found |
| *Cog3* | Cog3 | FBgn0031536 | Cytosol ER  Golgi | Conserved oligomeric Golgi (COG) complex, Protein transporter, dsRNA transport, ER to Golgi vesicle-mediated transport | COG3 | No disease terms found |
| *Amph* | Amphiphysin | FBgn0027356 | Cytosol Synaptic vesicles | Phospholipid binding, Neurotransmitter secretion, Synaptic vesicle endocytosis | BIN1 | Alzheimer's disease, Myopathy |
| *CG11819* | Unc-13-4B, Unc-13 ortholog 4B | FBgn0266719 | Cytosol Synaptic vesicles | Neurotransmitter secretion, Synaptic vesicle priming | UNC13D | Hemophagocytic lymphohistiocytosis |
| *lap* | like-AP180 | FBgn0086372 | Cytosol Synaptic vesicles | Clathrin binding, Phosphatidylinositol binding, Synaptic vesicle transport, Positive regulation of clathrin-mediated endocytosis | PICALM | Alzheimer's disease, Tourette syndrome, Leukemia, acute myeloid |
| *stnB* | stoned B | FBgn0016975 | Cytosol Synaptic vesicles | Protein binding, Synaptic vesicle coating, Neurotransmitter secretion, Synaptic vesicle endocytosis | STON2 | Obesity-related traits |
| *Sap47* | Synapse-associated protein 47kD | FBgn0013334 | Cytosol Synaptic vesicles | uknown | DNAJC13 | Parkinson Disease |
| *Sec5* | Sec5 ortholog (S. cerevisiae) | FBgn0266670 | Cytosol C/plasmic vesicles | Exocyst complex component, Rab GTPase binding | EXOC2 | Basal cell carcinoma, Black vs. blond hair color, Black vs. red hair color, Dental caries, Freckles, Schizophrenia |
| *Sec6* | Sec6 ortholog (S. cerevisiae) | FBgn0266671 | Cytosol C/plasmic vesicles | Exocyst complex component, SNARE binding | EXOC3 | Ulcerative colitis |
| *Sec8* | Sec8 ortholog (S. cerevisiae) | FBgn0266672 | Cytosol C/plasmic vesicles | Exocyst complex component | EXOC4 | Alzheimer's disease |
| *Sec10* | Sec10 ortholog  (S. cerevisiae) | FBgn0266673 | Cytosol C/plasmic vesicles | Exocyst complex component, beta-catenin binding | EXOC5 | No disease terms found |
| *Exo70* | Exo70 ortholog  (S. cerevisiae) | FBgn0266667 | Cytosol C/plasmic vesicles | Exocyst complex component, Phosphatidylinositol-4,5-bisphosphate binding, Beta-catenin binding | EXOC7 | No disease terms found |
| *Exo84* | Exo84 ortholog  (S. cerevisiae) | FBgn0266668 | Cytosol C/plasmic vesicles | Exocyst complex component | EXOC8 | No disease terms found |
| *mub* | mushroom-body expressed | FBgn0262737 | Nucleus Cytosol | Poly(C) RNA binding, Regulation of alternative mRNA splicing, via spliceosome | PCBP3 | Insulin resistance/response |
| *Neos* | Neosin | FBgn0024542 | Nucleus Cytosol | Nucleotide binding, Nucleic acid binding, mRNA binding, mRNA splicing, via spliceosome | NCOA5 | Multiple sclerosis |
| *emb* | embargoed | FBgn0020497 | Nucleus  Cytosol | exportin involved in protein export from the nucleus | EEF1A2 | Epileptic encephalopathy,  Mental retardation |
| *Runbp11*  *(Impβ11)* | Importin beta11 | FBgn0284254 | Nucleus  Cytosol | Ran GTPase binding | XPO1 | Primary Mediastinal Large B-Cell Lymphoma |
| *Ranbp16* | Ranbp16 | FBgn0053180 | Nuclear envelope | Importin-beta, Nuclear export signal receptor activity, Ran GTPase binding | RANBP17 | Visceral fat |
| *Kap-α1* | karyopherin α1 | FBgn0024889 | Nuclear envelope | Protein transmembrane transporter, Protein import into nucleus | KPNA1 KPNA5 KPNA3 KPNA7 | Calcium levels, Telomere length, Obesity-related traits, Ulcerative colitis |
| *Snr1* | Snf5-related 1 | FBgn0011715 | Nucleus | SWI/SNF chromatin-remodeling complex, SET domain binding, Transcription coactivator, Protein binding | SMARCB1 | IgG glycosylation, Mental retardation, Rhabdoid tumors |
| *Ssb-c31a* | Single stranded-binding protein c31A | FBgn0015299 | Nucleus | Single-stranded DNA binding, Transcription coactivator | SUB1 | No disease terms found |
| *osa* | osa | FBgn0261885 | Nucleus | Brahma complexes component, Transcription coactivator, DNA binding | ARID1A ARID1B | Alzheimer's disease biomarkers, Pancreatic cancer, Uric acid levels, Mental retardation |
| *mor* | moira | FBgn0002783 | Nucleus | Brahma complexes component, Protein binding, Transcription coactivator, DNA binding | SMARCC2 SMARCC1 | No disease terms found |
| *Not1* | Not1 | FBgn0085436 | Nucleus | CCR4-NOT transcription complex, Protein binding, Poly(A)-specific ribonuclease | HSD3B1-2 | Lipoid Congenital Adrenal Hyperplasia and Testicular Leydig Cell Tumor |
| *CG9932* | CG9932 | FBgn0262160 | Nucleus | unknown | CNOT1 | QT interval |
| *Tom70* | Trans-locase of outer membrane 70 | FBgn0032397 | Mitochon-dria | P-P-bond-hydrolysis-driven protein transmembrane transporter, Protein targeting to mitochondrion | TOMM70A | No disease terms found |
| *CG7382* | CG7382 | FBgn0031708 | Mitochon-dria | Mitochondrial import inner membrane translocase, Protein import into mitochondrial matrix | RUFY2 | Dyskeratosis congenita |
| *Pisd* | Phospha-tidylserine decarboxy-lase | FBgn0026576 | Mitochon-dria | Phosphatidylserine decarboxylase | TIMM21 | No disease terms found |
| *prom* | prominin | FBgn0259210 | - | unknown | PROM1 | Dental caries, Cone-rod dystrophy 12, Macular dystrophy, Retinitis pigmentosa, Stargardt disease |
| *CG30492* | CG43341 | FBgn0263046 | - | unknown | No ortholog found | - |
| *rogdi* | rogdi | FBgn0036697 | - | unknown | ROGDI | Kohlschutter-Tonz syndrome |
| *CG3662* | CG3662 | FBgn0031285 | - | unknown | ITM2A ITM2B | Height, Dementia |
| *CG14299* | CG14299 | FBgn0038651 | - | unknown | EPG5 | Vici syndrome |
| *CG3262* | CG3262 | FBgn0032986 | - | Nucleotide binding, ATP binding | NUBPL | Brain lesion load, Response to iloperidone treatment (QT prolongation), Complex I, mitochondrial respiratory chain deficiency |
| *kibra* | kibra ortholog | FBgn0262127 | - | Protein binding | WWC1-3 | Periodontal microbiota, Memory, Height |
| *Hpr1* | Hpr1 | FBgn0037382 | - | unknown | THOC1 | Bipolar disorder and major depressive disorder |
| *CG14647* | CG14647 | FBgn0037244 | - | unknown | KCTD9 | No disease terms found |
| *Bub3* | Bub3 | FBgn0025457 | - | unknown | BUB3 | No disease terms found |
| *Rrp40* | Rrp40 | FBgn0260648 | - | RNA binding | EXOSC3 | Pontocerebellar hypoplasia |
| *CG9588* | CG9588 | FBgn0038166 | - | unknown | PSMD9 | No disease terms found |
| *CG12333* | CG12333 | FBgn0038617 | - | unknown | WDR37 | Chronic kidney disease |
| *CG17765* | CG17765 | FBgn0033529 | - | EF-Hand 1, Calcium-binding site | PEF1 | No disease terms found |
| *CG5599* | CG5599 | FBgn0030612 | - | Dihydrolipoamide branched chain acyltransferase | DBT | Maple syrup urine disease |
| *CG5844* | CG5844 | FBgn0038049 | - | Dodecenoyl-CoA delta-isomerase | CDY1 | No disease terms found |
| *CG5608* | CG5608 | FBgn0038058 | - | unknown | VAC14 | No disease terms found |
| *CG31122* | CG31122 | FBgn0051122 | - | unknown | C2orf69 | No disease terms found |
| *CG7949* | galla-2 | FBgn0036107 | - | unknown | FAM96B | No disease terms found |
| *CG6617* | CG6617 | FBgn0030944 | - | unknown | GID8 | No disease terms found |
| *Ac3* | Ac3 | FBgn0023416 | - | Adenylate cyclase | ADCY3 | Body mass and height, Inflammatory bowel disease, Type 1 diabetes |
| *Scox* | Synthesis of cyto-chrome c oxidase | FBgn0262467 | - | Cytochrome-c oxidase, Copper chaperone | SCO1-2 | Malaria, Hepatic failure, Neurologic disorder, Multiple sclerosis, Cardioencephalomyopathy |
| *CG1513* | CG1513 | FBgn0033463 | - | Oxysterol binding | CAPN | Spastic paraplegia 76 (SPG76) |
| *Naxd* | NAD(P)HX dehydratase | FBgn0036848 | - | ADP(ATP)-dependent NAD(P)H-hydrate dehydratase | NAXD | Encephalopathy |
| *CG43340* | CG43340 | FBgn0263077 | - | unknown | No ortholog found | No disease terms found |
| *CG31064* | CG31064 | FBgn0051064 | - | Zinc finger, FYVE/PHD-type | PISD | Central Nervous System Origin Vertigo and Syndromic X-Linked Intellectual Disability Cabezas Type |
| *CG7724* | CG7724 | FBgn0036698 | - | 3-beta-hydroxy-Delta(5)-steroid dehydrogenase | No ortholog found | No disease terms found |
| *cha* | chaff | FBgn0000302 | - | unknown | No ortholog found | No disease terms found |
