# Supplementary material for: Exocyst-mediated membrane trafficking of the lissencephaly-associated ECM receptor dystroglycan is required for proper brain compartmentalization
Source: eLife. 2021 Feb 23;10:e63868. doi: 10.7554/eLife.63868 (PMC7929561; doi:10.7554/eLife.63868)
Supplement: Supplementary file 3. — For human disease-association enrichment analysis, the entry of human orthologs of identified Dg-interacting components (Table S2) was examined with Comparative Toxicogenomics Database (CTD) Disease Tool http://ctdbase.org/tools. The corrected threshold value of p<0.001 was used. [file elife-63868-supp3.docx]

**Supplementary File 3. Human disease enrichment in the Dg neuronal interactome network**

| Disease Name | Disease  ID | Disease Categories | P-value | Corrected P-value | Annotated Genes Quantity | Genome Frequency |
| --- | --- | --- | --- | --- | --- | --- |
| Congenital, Hereditary, and Neonatal Diseases and Abnormalities | MESH:D009358 | Congenital, Hereditary, and Neonatal Diseases and Abnormalities | 4.93e-10 | 2.82e-7 | 29 | 2877/43622 genes: 6.60% |
| Neoplasms | MESH:D009369 | Cancer | 2.50e-9 | 1.44e-6 | 32 | 3715/43622 genes: 8.52% |
| Genetic Diseases, Inborn | MESH:D030342 | Genetic disease (inborn) | 6.05e-9 | 3.47e-6 | 24 | 2233/43622 genes: 5.12% |
| Nervous System Diseases | MESH:D009422 | Nervous system disease | 7.79e-9 | 4.46e-6 | 26 | 2643/43622 genes: 6.06% |
| Neoplasms by Site | MESH:D009371 | Cancer | 1.50e-8 | 8.62e-6 | 27 | 2931/43622 genes: 6.72% |
| Mental Disorders | MESH:D001523 | Mental disorder | 3.52e-8 | 2.01e-5 | 18 | 1379/43622 genes: 3.16% |
| Pathological Conditions, Signs and Symptoms | MESH:D013568 | Pathological Conditions, Signs and Symptoms | 8.24e-8 | 4.72e-5 | 28 | 3399/43622 genes: 7.79% |
| Neoplasms by Histologic Type | MESH:D009370 | Cancer | 3.60e-7 | 2.07e-4 | 21 | 2172/43622 genes: 4.98% |
| Coffin-Siris syndrome | MESH:C536436 | Congenital abnormality  Mental disorder  Musculoskeletal disease  Nervous system disease | 6.70e-7 | 3.84e-4 | 3 | 7/43622 genes: 0.02% |
| Male Urogenital Diseases | MESH:D052801 | Urogenital disease (male) | 7.11e-7 | 4.07e-4 | 17 | 1513/43622 genes: 3.47% |
| Rectal Diseases | MESH:D012002 | Digestive system disease | 1.04e-6 | 5.94e-4 | 8 | 280/43622 genes: 0.64% |
| Digestive System Neoplasms | MESH:D004067 | Cancer  Digestive system disease | 1.66e-6 | 9.51e-4 | 16 | 1431/43622 genes: 3.28% |

For human disease-association enrichment analysis, the entry of human orthologs of identified Dg-interacting components (Table S2) was examined with Comparative Toxicogenomics Database (CTD) Disease Tool <http://ctdbase.org/tools>. The corrected threshold value of P<0.001 was used.
