# Supplementary material for: Exocyst-mediated membrane trafficking of the lissencephaly-associated ECM receptor dystroglycan is required for proper brain compartmentalization
Source: eLife. 2021 Feb 23;10:e63868. doi: 10.7554/eLife.63868 (PMC7929561; doi:10.7554/eLife.63868)
Supplement: Supplementary file 4. [file elife-63868-supp4.docx]

**Supplementary File 4. Functional enrichments in the Dg neuronal interactome network based on cellular component (GO) terms**

| #term ID | Term description | Observed gene count | Background gene count | False discovery rate | Matching proteins in the network |
| --- | --- | --- | --- | --- | --- |
| GO:0044464 | cell part | 83 | 6736 | 1.29e-08 | Ac3, Amph, Apoltp, Bub3, CG10226, CG1090, CG11306, CG12333, CG13185, CG32850, CG3626, CG3662, CG3689, CG4538, CG5599, CG5608, CG5853, CG5991, CG7382, CG7949, CG9588, CG9932, CSN4, CSN5, CalpA, Cog3, Cp190, Crag, Cul1, Cul3, Cul4, Dg, Eif2b, Exo70, Exo84, F2, Hem, Hpr1, Kap-alpha1, MESK2, Not1, Pka-C1, Pka-R1, Pkc98E, PlexA, RanGAP, Ranbp11, Ranbp16, Rme-8, Rrp40, Rrp46, Scox, Sec10, Sec5, Sec6, Sec8, Snr1, Srp54k, Ssb-c31a, Tom70, Uev1A, Unc-13-4B, bchs, boss, bur, dor, emb, eys, faf, for, garz, igl, kcc, kibra, lap, mor, msi, mub, osa, prom, stnB, vimar, zw2 |
| GO:0000145 | exocyst | 6 | 8 | 3.74e-08 | Exo70, Exo84, Sec10, Sec5, Sec6, Sec8 |
| GO:0005622 | intra-cellular | 75 | 5815 | 3.74e-08 | Ac3, Amph, Bub3, CG11306, CG12333, CG13185, CG32850, CG3626, CG3662, CG3689, CG4538, CG5599, CG5608, CG5991, CG7382, CG7949, CG9588, CG9932, CSN4, CSN5, CalpA, Cog3, Cp190, Crag, Cul1, Cul3, Cul4, Dg, Eif2b, Exo70, Exo84, F2, Hem, Hpr1, Kap-alpha1, MESK2, Not1, Pka-C1, Pka-R1, Pkc98E, RanGAP, Ranbp11, Ranbp16, Rme-8, Rrp40, Rrp46, Scox, Sec10, Sec5, Sec6, Sec8, Snr1, Srp54k, Ssb-c31a, Tom70, Uev1A, Unc-13-4B, bchs, boss, bur, dor, emb, faf, for, garz, igl, kibra, lap, mor, msi, mub, osa, stnB, vimar, zw2 |
| GO:0032991 | protein-containing complex | 47 | 2565 | 3.74e-08 | Ac3, Bub3, CG10226, CG12333, CG13185, CG32850, CG3689, CG5599, CG5608, CG7382, CG7949, CG9588, CSN4, CSN5, Cog3, Cp190, Cul1, Cul3, Cul4, Dg, Eif2b, Exo70, Exo84, F2, Hem, Hpr1, Kap-alpha1, Not1, Pka-R1, Ranbp16, Rrp40, Rrp46, Sec10, Sec5, Sec6, Sec8, Snr1, Srp54k, Ssb-c31a, Tom70, Uev1A, bur, dor, emb, kibra, mor, osa |
| GO:0044424 | intra-cellular part | 74 | 5758 | 3.91e-08 | Ac3, Amph, Bub3, CG11306, CG12333, CG13185, CG32850, CG3626, CG3662, CG3689, CG4538, CG5599, CG5608, CG5991, CG7382, CG7949, CG9588, CG9932, CSN4, CSN5, CalpA, Cog3, Cp190, Crag, Cul1, Cul3, Cul4, Dg, Eif2b, Exo70, Exo84, F2, Hem, Hpr1, Kap-alpha1, MESK2, Not1, Pka-C1, Pka-R1, RanGAP, Ranbp11, Ranbp16, Rme-8, Rrp40, Rrp46, Scox, Sec10, Sec5, Sec6, Sec8, Snr1, Srp54k, Ssb-c31a, Tom70, Uev1A, Unc-13-4B, bchs, boss, bur, dor, emb, faf, for, garz, igl, kibra, lap, mor, msi, mub, osa, stnB, vimar, zw2 |
| GO:0005737 | cytoplasm | 57 | 3684 | 4.07e-08 | Ac3, Amph, CG11306, CG3626, CG3662, CG4538, CG5599, CG5608, CG5991, CG7382, CG7949, CG9588, CSN4, CSN5, CalpA, Cog3, Cp190, Crag, Cul3, Dg, Eif2b, Exo70, Exo84, F2, Hem, Kap-alpha1, MESK2, Not1, Pka-C1, RanGAP, Ranbp11, Ranbp16, Rme-8, Rrp40, Rrp46, Scox, Sec10, Sec5, Sec6, Sec8, Srp54k, Tom70, Uev1A, Unc-13-4B, bchs, boss, dor, emb, faf, for, garz, igl, kibra, lap, stnB, vimar, zw2 |
| GO:0099023 | tethering complex | 8 | 42 | 9.90e-08 | Cog3, Exo70, Exo84, Sec10, Sec5, Sec6, Sec8, dor |
| GO:0044444 | cyto-plasmic part | 44 | 2614 | 9.67e-07 | Ac3, CG11306, CG3626, CG3662, CG4538, CG5599, CG5608, CG5991, CG7382, CG7949, CSN4, Cog3, Cp190, Crag, Dg, Eif2b, Exo70, Exo84, F2, Hem, Kap-alpha1, Not1, RanGAP, Ranbp11, Rme-8, Rrp40, Rrp46, Scox, Sec10, Sec5, Sec6, Sec8, Srp54k, Tom70, Unc-13-4B, bchs, boss, dor, garz, igl, kibra, lap, stnB, vimar |
| GO:0044448 | cell cortex part | 8 | 83 | 9.77e-06 | Crag, Exo70, Exo84, Sec10, Sec5, Sec6, Sec8, kibra |
| GO:0005938 | cell cortex | 9 | 151 | 7.10e-05 | Crag, Exo70, Exo84, Sec10, Sec5, Sec6, Sec8, igl, kibra |
| GO:0071944 | cell periphery | 24 | 1225 | 0.00025 | Ac3, Amph, Apoltp, CG10226, CG1090, CG3662, CG5853, Crag, Dg, Exo70, Exo84, Pka-C1, Pkc98E, PlexA, Sec10, Sec5, Sec6, Sec8, boss, for, igl, kcc, kibra, zw2 |
| GO:0035060 | brahma complex | 3 | 12 | 0.0033 | Snr1, mor, osa |
| GO:0016028 | rhabdo-mere | 4 | 35 | 0.0035 | Sec5, Sec6, Sec8, prom |
| GO:0005886 | plasma membrane | 19 | 1085 | 0.0074 | Ac3, Amph, Apoltp, CG10226, CG1090, CG3662, CG5853, Crag, Dg, Pka-C1, Pkc98E, PlexA, Sec5, Sec8, boss, for, kcc, kibra, zw2 |
| GO:0045202 | synapse | 8 | 234 | 0.0074 | Amph, Dg, Sap47, Sec8, Unc-13-4B, bchs, dor, stnB |
| GO:0043226 | organelle | 51 | 4694 | 0.0139 | Bub3, CG11306, CG12333, CG13185, CG3626, CG3662, CG3689, CG4538, CG5599, CG5608, CG5991, CG7382, CG9588, CG9932, CSN4, CSN5, CalpA, Cog3, Cp190, Crag, Cul1, Cul4, Dg, Hpr1, Kap-alpha1, Not1, Ranbp11, Ranbp16, Rme-8, Rrp40, Rrp46, Scox, Sec5, Snr1, Ssb-c31a, Tom70, Uev1A, Unc-13-4B, bchs, boss, bur, dor, emb, eys, garz, lap, mor, msi, mub, osa, stnB |
| GO:0043227 | membrane-bounded organelle | 46 | 4110 | 0.0155 | Bub3, CG11306, CG12333, CG13185, CG3626, CG3662, CG3689, CG4538, CG5599, CG5608, CG5991, CG7382, CG9588, CG9932, CSN4, CSN5, Cog3, Cp190, Crag, Cul1, Cul4, Hpr1, Kap-alpha1, Ranbp11, Ranbp16, Rme-8, Rrp40, Rrp46, Scox, Sec5, Snr1, Ssb-c31a, Tom70, Uev1A, Unc-13-4B, bchs, boss, dor, emb, garz, lap, mor, msi, mub, osa, stnB |
| GO:0043229 | intra-cellular organelle | 50 | 4624 | 0.0168 | Bub3, CG11306, CG12333, CG13185, CG3626, CG3662, CG3689, CG4538, CG5599, CG5608, CG5991, CG7382, CG9588, CG9932, CSN4, CSN5, CalpA, Cog3, Cp190, Crag, Cul1, Cul4, Dg, Hpr1, Kap-alpha1, Not1, Ranbp11, Ranbp16, Rme-8, Rrp40, Rrp46, Scox, Sec5, Snr1, Ssb-c31a, Tom70, Uev1A, Unc-13-4B, bchs, boss, bur, dor, emb, garz, lap, mor, msi, mub, osa, stnB |
| GO:0012505 | endo-membrane system | 16 | 924 | 0.0191 | CG11306, CG3662, Cog3, Crag, Kap-alpha1, Ranbp11, Ranbp16, Rme-8, Sec5, Unc-13-4B, boss, dor, emb, garz, lap, stnB |
| GO:0016020 | membrane | 32 | 2553 | 0.0195 | Ac3, Amph, Apoltp, CG10226, CG1090, CG11306, CG3662, CG5608, CG5853, CG7382, Cog3, Crag, Dg, Pka-C1, Pkc98E, PlexA, Rme-8, Scox, Sec5, Sec6, Sec8, Tom70, boss, dor, emb, for, igl, kcc, kibra, lap, prom, zw2 |
| GO:0031982 | vesicle | 9 | 365 | 0.0215 | CG3662, Crag, Rme-8, Sec5, Unc-13-4B, boss, dor, lap, stnB |
| GO:1904949 | ATPase complex | 4 | 66 | 0.0215 | CG10226, Snr1, mor, osa |
| GO:0045178 | basal part of cell | 3 | 32 | 0.0240 | Apoltp, Dg, Sec8 |
| GO:0000176  GO:0000177 | RNase complex | 2 | 8 | 0.0250 | Rrp40, Rrp46 |
| GO:0005801 | cis-Golgi network | 2 | 8 | 0.0250 | Cog3, garz |
| GO:0008180 | COP9 signalosome | 2 | 10 | 0.0320 | CSN4, CSN5 |
| GO:0016586 | RSC-type complex | 2 | 10 | 0.0320 | Snr1, mor |
| GO:0031410 | cyto-plasmic vesicle | 8 | 324 | 0.0320 | Crag, Rme-8, Sec5, Unc-13-4B, boss, dor, lap, stnB |
| GO:0005902 | microvillus | 2 | 11 | 0.0347 | boss, prom |
| GO:0009925 | basal plasma membrane | 2 | 11 | 0.0347 | Dg, Sec8 |
| GO:0005643 | nuclear pore | 3 | 44 | 0.0389 | Kap-alpha1, Ranbp16, emb |
| GO:1902494 | catalytic complex | 14 | 858 | 0.0389 | CG10226, CG32850, CG5599, CG5608, CG9588, Cul1, Cul3, Cul4, Rrp40, Rrp46, Snr1, Uev1A, mor, osa |
