# Supplementary material for: Exocyst-mediated membrane trafficking of the lissencephaly-associated ECM receptor dystroglycan is required for proper brain compartmentalization
Source: eLife. 2021 Feb 23;10:e63868. doi: 10.7554/eLife.63868 (PMC7929561; doi:10.7554/eLife.63868)
Supplement: Supplementary file 5. — STRING database (https://string-db.org) was used to identify functional networks with the interaction score of high confidence (0.700). Interacting protein are clustered by the MCL with the inflation parameter (3). For the schematic representation of the obtained interaction network, see Figure 4D. [file elife-63868-supp5.docx]

**Supplementary File 5. Dg-associated components placed into the protein-interaction network identified by Markov Cluster Algorithm (MCL)**

| function | cluster number | cluster color | gene count | protein name | protein description |
| --- | --- | --- | --- | --- | --- |
| Exocyst complex/ vesicle | 1 | Red | 7 | Sec10 | Exocyst complex component 5; Component of the exocyst complex involved in the docking of exocytic vesicles with fusion sites on the plasma membrane |
|  | 1 | Red | 7 | Exo70 | Exocyst complex component 7; Required for exocytosis. Thought to function in intracellular vesicle targeting and docking before SNARE complex formation |
|  | 1 | Red | 7 | Sec5 | Exocyst complex component 2; Component of the exocyst complex involved in the docking of exocytic vesicles with fusion sites on the plasma membrane |
|  | 1 | Red | 7 | Cog3 | Conserved oligomeric Golgi complex subunit 3; Involved in ER-Golgi transport |
|  | 1 | Red | 7 | Sec8 | Exocyst complex component 4; Component of the exocyst complex involved in the docking of exocytic vesicles with fusion sites on the plasma membrane. Involved in regulation of synaptic microtubule formation, and also regulation of synaptic growth and glutamate receptor trafficking. Does not appear to be required for basal neurotransmission |
|  | 1 | Red | 7 | Exo84 | annotation not available |
|  | 1 | Red | 7 | Sec6 | Exocyst complex component 3; Component of the exocyst complex involved in the docking of exocytic vesicles with fusion sites on the plasma membrane |
| Golgi/ER transport | 2 | Light Green | 4 | emb | Exportin-1; Receptor for the leucine-rich nuclear export signal (NES). Binds cooperatively to the NES on its target protein and to the small GTPase Ran in its active GTP-bound form. Required for the function or maintenance of certain tissues such as brain and gut. Involved in the export of dl from the nucleus to the cytoplasm; Belongs to the exportin family |
|  | 2 | Light Green | 4 | Ranbp16 | Ran-binding protein 16; May function as a nuclear transport receptor |
|  | 2 | Light Green | 4 | Kap-alpha1 | Importin subunit alpha; Protein transmembrane transporter activity. It is involved in the biological process described with: protein import into nucleus; spermatogenesis; Belongs to the importin alpha family |
|  | 2 | Light Green | 4 | RanGAP | Ran GTPase-activating protein; GTPase activator for the nuclear Ras-related regulatory protein Ran, converting it to the putatively inactive GDP-bound state (By similarity). Trans-acting factor necessary for meiotic distortion. Distortion is only seen in individuals that carry the RanGAP tandem duplication and express a RanGAP truncated protein. Binding of truncated RanGAP product to the Responder(RSP) locus initiates events that lead to sperm dysfunction |
|  | 10 | Brown | 2 | garz | Gartenzwerg, isoform B; Gartenzwerg (Garz) is a guanine nucleotide exchange factor localized to the cis-Golgi apparatus. Garz roles include membrane and protein trafficking between the Golgi apparatus and the endoplasmic reticulum. Garz is required for epithelial morphogenesis in the embryo |
| RNA processing | 10 | Brown | 2 | Not1 | Not1, isoform C; Protein binding. It is involved in the biological process described with: mRNA catabolic process; muscle organ development; mitotic G2 DNA damage checkpoint; dendrite morphogenesis; neurogenesis; nuclear-transcribed mRNA poly(A) tail shortening; negative regulation of translation |
|  | 5 | Medium Purple | 3 | CG3689 | CG3689, isoform C; mRNA binding; hydrolase activity. It is involved in the biological process described with: mRNA cleavage; mRNA polyadenylation |
|  | 5 | Medium Purple | 3 | mub | Mushroom-body expressed, isoform K; poly(C) RNA binding. It is involved in the biological process described with: regulation of alternative mRNA splicing, via spliceosome; thermosensory behavior |
|  | 5 | Medium Purple | 3 | Hpr1 | Hpr1 protein; It is involved in the biological process described with: mRNA export from nucleus in response to heat stress; mRNA export from nucleus; signal transduction |
|  | 14 | Green Yellow | 2 | Rrp46 | FI06805p; 3'-5'-exoribonuclease activity. It is involved in the biological process described with: regulation of gene expression; mRNA processing |
|  | 14 | Green Yellow | 2 | Rrp40 | Ribosomal RNA processing 40, isoform A; 3'-5'-exoribonuclease activity; RNA binding. It is involved in the biological process described with: regulation of gene expression; rRNA processing |
| (de)Phosphorylation | 3 | Medium Sea Green | 4 | Pka-C1 | Protein kinase, cAMP-dependent, catalytic subunit 1 is a serine/threonine kinase that contributes to axis specification, rhythmic behavior and synaptic transmission |
|  | 3 | Medium Sea Green | 4 | Pka-R1 | Cyclic nucleotide binding; cAMP-dependent protein kinase regulator activity. It is involved in the biological process described with: regulation of protein phosphorylation; actin filament organization; positive regulation of feeding behavior; cAMP-mediated signaling; response to ethanol; molting cycle, chitin-based cuticle; olfactory learning; neuromuscular synaptic transmission; oocyte microtubule cytoskeleton polarization |
|  | 3 | Medium Sea Green | 4 | Ac3 | Ac3, isoform A; Ac3 is an adenylate cyclase that is coupled to the receptor Pdfr via Gs signaling in the Morning circadian pacemakers |
|  | 3 | Medium Sea Green | 4 | for | cGMP-dependent protein kinase, isozyme 2 forms cD4/T1/T3A/T3B; Foraging (For) is a serine/threonine kinase and a member of the Protein Kinase G (PKG) family. For roles include feeding, locomotion, metabolism, development, olfactory habituation, learning and memory, stress, social behavior, as well as Malpighian tubule, heart, muscle and synaptic function |
| Protein ubiquitination/degradation | 4 | Cyan | 4 | Uev1A | Ubiquitin-conjugating enzyme variant 1A (Uev1A) is a conserved protein that contributes to ubiquitin protein ligase activity, but not catalytically, since it lacks the conserved cysteine residue essential for protein ubiquitination. Uev1A regulates genomic integrity, IMD pathway-mediated innate immunity, JNK-pathway mediated cell death and tumor invasion |
|  | 4 | Cyan | 4 | Cul1 | Cullin homolog 1; Core component of multiple SCF (SKP1-CUL1-F-box protein) E3 ubiquitin-protein ligase complexes which mediate the ubiquitination of proteins involved in cell cycle progression, signal transduction and transcription. In the SCF complex, serves as a rigid scaffold that organizes the SKP1-F-box protein and RBX1 subunits. May contribute to catalysis through positioning of the substrate and the ubiquitin-conjugating enzyme. During early metamorphosis, part of the SCF-slmb complex that negatively regulates the InR/PI3K/TOR pathway to activate the pruning of unnecessary larva axons |
|  | 4 | Cyan | 4 | Cul3 | Cullin 3, isoform F; Ubiquitin-protein transferase activity; ubiquitin protein ligase binding; Belongs to the cullin family |
|  | 4 | Cyan | 4 | Cul4 | Cullin 4 is the molecular scaffold for the CRL4 E3 ubiquitin ligase complex, which catalyzes the ubiquitylation and subsequent destruction of proteins that function in cell growth and proliferation as well as transcription, replication and repair of the genome; Belongs to the cullin family |
|  | 11 | Dark Golden Rod | 2 | CSN5 | COP9 signalosome complex subunit 5; Probable protease subunit of the COP9 signalosome complex (CSN), a complex involved in various cellular and developmental processes. The CSN complex is an essential regulator of the ubiquitin (Ubl) conjugation pathway by mediating the deneddylation of the cullin subunits of the SCF-type E3 ligase complexes, leading to decrease the Ubl ligase activity of SCF. In the complex, it probably acts as the catalytic center that mediates the cleavage of Nedd8 from cullins. It however has no metalloprotease activity by itself and requires the other subunits |
|  | 11 | Dark Golden Rod | 2 | CSN4 | COP9 signalosome complex subunit 4; Component of the COP9 signalosome complex (CSN), a complex involved in various cellular and developmental processes. The CSN complex is an essential regulator of the ubiquitin (Ubl) conjugation pathway by mediating the deneddylation of the cullin subunits of the SCF-type E3 ligase complexes, leading to decrease the Ubl ligase activity of SCF. The CSN complex plays an essential role in oogenesis and embryogenesis and is required for proper photoreceptor R cell differentiation and promote lamina glial cell migration or axon targeting. |
|  | 15 | Green | 2 | Bub3 | Bub3, isoform A; Bub3 functions in the "spindle assembly checkpoint (SAC)" pathway. This mitotic checkpoint pathway inhibits the APC/C complex and hence the transition from metaphase to anaphase, as long as there are free kinetochores that are not yet attached to the mitotic spindle in the cell. Bub3 is localized at the kinetochore during mitosis with maximal levels observed at free kinetochores. Together with other SAC proteins it forms mitotic checkpoint complexes that bind and inhibit the APC/C |
|  | 15 | Green | 2 | CG9588 | 26S proteasome non-ATPase regulatory subunit 9; Acts as a chaperone during the assembly of the 26S proteasome, specifically of the base subcomplex of the PA700/19S regulatory complex (RC) |
| Photoreceptor development | 6 | Orchid | 5 | Dg | Dystroglycan, isoform D; Dystroglycan is a major non-integrin ECM receptor that connects the extracellular matrix to the actin cytoskeleton. It regulates animal survival and temperature preference, muscle integrity, myotendinous and neuromuscular junction formation and function, nervous system development, axon pathfinding, rhabdomere differentiation, neuronal stem cell division and epithelial polarity |
|  | 6 | Orchid | 5 | eys | Protein eyes shut; Essential for the formation of matrix-filled interrhabdomeral space: critical for the formation of epithelial lumina in the retina. Acts together with prominin (prom) and the cell adhesion molecule chaoptin (chp) to choreograph the partitioning of rhabdomeres into an open system |
|  | 6 | Orchid | 5 | prom | Prominin, isoform D; Prominin (Prom) is an evolutionary conserved five transmembrane protein. Prom is required for the correct positioning and formation of photoreceptor rhabdomeres |
|  | 6 | Orchid | 5 | vimar | Visceral mesodermal armadillo-repeats is involved in regulation of muscle homeostasis and rhabdomere development. Its expression in the visceral mesoderm is controlled by bap |
|  | 6 | Orchid | 5 | kibra | Protein kibra; Regulator of the Hippo/SWH (Sav/Wts/Hpo) signaling pathway, a signaling pathway that plays a pivotal role in organ size control and tumor suppression by restricting proliferation and promoting apoptosis. The core of this pathway is composed of a kinase cascade wherein Hippo (Hpo), in complex with its regulatory protein Salvador (Sav), phosphorylates and activates Warts (Wts) in complex with its regulatory protein Mats, which in turn phosphorylates and inactivates the Yorkie (Yki) oncoprotein. Kibra acts synergistically along with Ex and Mer to regulate the Hippo signaling |
| Chromatin remodelling | 9 | Pale Violet Red | 3 | osa | Trithorax group protein osa; Trithorax group (trxG) protein required for embryonic segmentation, development of the notum and wing margin, and photoreceptor differentiation. Required for the activation of genes such as Antp, Ubx and Eve. Binds to DNA without specific affinity, suggesting that it is recruited to promoters by promoter-specific proteins. Essential component of the Brahma complex, a multiprotein complex which is the equivalent of the yeast SWI/SNF complex and acts by remodeling the chromatin by catalyzing an ATP-dependent alteration in the structure of nucleosomal DNA |
|  | 9 | Pale Violet Red | 3 | Snr1 | Snf5-related 1; Protein binding; SET domain binding; transcription coactivator activity |
|  | 9 | Pale Violet Red | 3 | mor | Brahma associated protein 155 kDa; DNA binding; protein binding; chromatin binding; transcription coactivator activity |
| Synaptic signaling | 8 | Pink | 3 | Amph | Amphiphysin, isoform A; Phospholipid binding. It is involved in the biological process described with: exocytosis; protein localization; rhabdomere development; synaptic vesicle endocytosis; endocytosis; neurotransmitter secretion; rhabdomere membrane biogenesis; regulation of muscle contraction |
|  | 8 | Pink | 3 | stnB | Protein stoned-B; Adapter protein involved in endocytic recycling of synaptic vesicles membranes. May act by mediating the retrieval of synaptotagmin protein Syt from the plasma membrane, thereby facilitating the internalization of multiple synaptic vesicles from the plasma membrane |
|  | 8 | Pink | 3 | lap | Phosphatidylinositol-binding clathrin assembly protein LAP; Assembly protein recruiting clathrin and adaptor protein complex 2 (AP2) to cell membranes at sites of coated-pit formation and clathrin-vesicle assembly. May be required to determine the amount of membrane to be recycled, possibly by regulating the size of the clathrin cage. Involved in AP2-dependent clathrin-mediated endocytosis at the neuromuscular junction; Belongs to the PICALM/SNAP91 family |
| Mitochondria targeting | 12 | Yellow | 2 | CG7382 | GM05057p; It is involved in the biological process described with: protein import into mitochondrial matrix |
|  | 12 | Yellow | 2 | Tom70 | Translocase of outer membrane 70, isoform A; P-P-bond-hydrolysis-driven protein transmembrane transporter activity. It is involved in the biological process described with: protein targeting to mitochondrion |
| Membrane function | 13 | Olive | 2 | uzip | Unzipped is a cell adhesion molecule that genetically interacts with CadN. Its biological function includes regulation of axon guidance |
|  | 13 | Olive | 2 | CG31122 | Uncharacterized protein, isoform A; It is involved in the biological process described with: lateral inhibition |
|  | 7 | Purple | 3 | CG17765 | GH27120p; Calcium ion binding |
|  | 7 | Purple | 3 | CalpA | Calpain-A; Calcium-regulated non-lysosomal thiol-protease. Involved in the organization of the actin-related cytoskeleton during embryogenesis |

STRING database (<https://string-db.org>) was used to identify functional networks with the interaction score of high confidence (0.700). Interacting protein are clustered by the MCL with the inflation parameter (3). For the schematic representation of the obtained interaction network, see Figure 4D.
